# Association of body mass index changes with short-term mortality risks in ICU patients with sepsis across different admission BMI states: analysis of the MIMIC-IV database

**DOI:** 10.3389/fimmu.2025.1698405

**Published:** 2025-10-24

**Authors:** Wei Liu, Wenfei Zeng, Zhenhua Huang, Qinghua Yuan

**Affiliations:** 1 Department of Emergency Medicine, The Huangpu People’s Hospital of Zhongshan, Zhongshan, China; 2 Department of Anesthesiology, Hunan Provincial People’s Hospital, The First Affiliated Hospital of Hunan Normal University, Changsha, China; 3 Department of Emergency Medicine, the First Affiliated Hospital of Shenzhen University, 7Shenzhen Second People’s Hospital, Shenzhen, China; 4 Department of Cardiology, the Seventh Affiliated Hospital of Sun Yat-sen University, Shenzhen, Guangdong, China

**Keywords:** sepsis, body-mass-index change, intensive care unit (ICU), 30-day mortality, MIMIC-IV database

## Abstract

**Background:**

Static body mass index (BMI) is a known predictor of mortality in sepsis; however, the prognostic value of dynamic BMI trajectories across various admission BMI states remains unclear. This study aimed to quantify the dose–response relationship between ICU-acquired BMI changes and 30-day mortality and to determine BMI-specific thresholds for risk stratification.

**Methods:**

This retrospective, multicenter cohort study analyzed 5,577 adult patients with sepsis from the MIMIC-IV database (2008–2022). To delineate the nature of the relationship between BMI change rate and 30-day mortality across distinct baseline BMI strata, we employed multivariable Cox proportional hazards regression coupled with restricted cubic splines. A two-segment linear regression model with a recursive algorithm was then applied to pinpoint inflection points for each BMI-defined subgroup.

**Results:**

Among 5,577 ICU patients with sepsis (mean age 66.5 ± 15.8 years; 57.4% male), 2,068 deaths (37.1%) occurred within 30 days. BMI change during the ICU stay ranged from −39% to +49%. After multivariable adjustment, each 1% increase in BMI change rate was associated with a 2% higher 30-day mortality (hazard ratio [HR] 1.02; 95% confidence interval [CI] 1.02–1.02; *p* < 0.001). However, in the underweight subgroup (<18.5 kg/m²), no significant association was observed (HR 0.99; 95% CI 0.98–1.00; *p* = 0.093). Restricted cubic spline analyses revealed BMI-specific inflection points: −2% in underweight, +4% in normal-weight (18.5–24.9 kg/m²), and −1% in overweight/obese (≥25 kg/m²) patients (p for nonlinearity < 0.001 for all). Dynamic BMI metrics significantly outperformed admission BMI in predicting 30-day mortality (*p* < 0.001).

**Conclusion:**

In critically ill ICU patients with sepsis, the relationship between BMI change (%) and 30-day mortality is nonlinear and varies across baseline BMI. Among patients with an admission BMI ≥18.5 kg/m², an increase in BMI during the ICU stay is associated with higher mortality risk, indicating that weight gain is deleterious. Conversely, in patients with an admission BMI <18.5 kg/m², a decline in BMI markedly amplifies the risk of death. Tailored and dynamic weight management strategies accounting for baseline BMI trajectories may therefore help mitigate sepsis-related mortality.

## 1 Introduction

Sepsis is a dysregulated host response to infection, leading to life-threatening organ dysfunction and accounting for approximately 20% of global deaths annually (1). Despite advances in sepsis management, mortality remains high, with short-term case fatality rates ranging from 15%–30% and one-year mortality exceeding 40% (2). Identifying modifiable prognostic factors is therefore critical to improving outcomes. Among these, nutritional status—commonly assessed via body mass index (BMI)—has emerged as a key determinant of sepsis survival, though its relationship with mortality is complex and context dependent (3).

A growing body of evidence supports a U-shaped or reverse J-shaped association between BMI and sepsis mortality. In a recent meta-analysis of 105,159 patients, overweight (BMI 25–29.9 kg/m²) and obese (BMI 30–39.9 kg/m²) individuals demonstrated 21% and 26% lower mortality odds, respectively, compared with normal-weight counterparts (4). Conversely, underweight patients (BMI <18.5 kg/m²) face markedly elevated risks. In a prospective cohort of 0.5 million Chinese adults, underweight was associated with a 2.42-fold higher long-term sepsis-related mortality (hazard ratio [HR] = 2.42; 95% confidence interval [CI] 2.07–2.84) (5). These findings underscore the “obesity paradox” in sepsis, wherein adipose tissue may confer metabolic reserves and anti-inflammatory effects (6).

However, prior studies predominantly relied on single-point BMI measurements at ICU admission, failing to capture dynamic changes during critical illness (7). ICU patients often experience rapid catabolism, fluid shifts, and sarcopenia, which may exacerbate mortality risks in underweight individuals (8). Recent analyses using the MIMIC-IV database suggest that BMI trajectories—rather than static values—may better predict outcomes. For instance, in patients with *Staphylococcus aureus* sepsis, each 1 kg/m² increase in BMI was associated with a 2.8% reduction in 28-day mortality (adjusted odds ratio [aOR] = 0.972; 95% CI 0.955–0.990) (9). Although prior MIMIC-IV analyses suggested that a static high BMI is associated with better short-term survival in sepsis (9), a recent multicenter retrospective cohort using eICU-CRD demonstrated that in-hospital BMI gain was linked to significantly higher ICU and in-hospital mortality (odds ratio [OR] 1.36 and 1.25, both *p* < 0.001) (10). This paradox indicates that (i) a single BMI measurement may be confounded by acute fluid shifts and resuscitation, failing to reflect true nutritional reserve, and (ii) only *dynamic* BMI trajectories (ICU admission → ICU discharge) can reveal the real association between weight change and death risk.

However, no systematic study has evaluated entire-course BMI change in sepsis and its relationship with short-term (30-day) mortality. Therefore, we built a sepsis cohort from MIMIC-IV and conducted a longitudinal analysis of BMI change versus death outcomes to quantify the relationship between BMI variation during hospitalization and 30-day mortality. Findings will inform evidence-based “precision BMI management” strategies—defining the optimal magnitude and target range—to improve early prognosis in patients with sepsis.

## 2 Methods

### 2.1 Study design and data source

This retrospective cohort analysis utilized the MIMIC-IV version 3.1 database, which integrates de-identified clinical data from intensive care units at Beth Israel Deaconess Medical Center between 2008 and 2022 (11). The dataset includes patient demographics, physiologic indicators, laboratory results, and clinical diagnoses. The study was designed and implemented in full accordance with the Strengthening the Reporting of Observational Studies in Epidemiology (STROBE) guidelines.

### 2.2 Study population

Data were extracted from the MIMIC-IV database (version 3.1) using PostgreSQL version 17.7 and structured query language (SQL). The original cohort comprised 11,726 adult patients who met all predefined criteria.

Inclusion criteria:

1. Age ≥18 years;
2. Fulfillment of the Sepsis-3.0 definition, i.e., suspected or confirmed infection with a Sequential Organ Failure Assessment (SOFA) score ≥2 (12);
3. Intensive Care Unit (ICU) length of stay ≥24 h;
4. First ICU admission for sepsis.

Exclusion criteria:

1. Only the first ICU stay was retained for patients with multiple admissions;
2. Missing admission or discharge weight;
3. Missing ICU or hospital length of stay;
4. Discrete values for weight changes unavailable.

**Figure 1:**
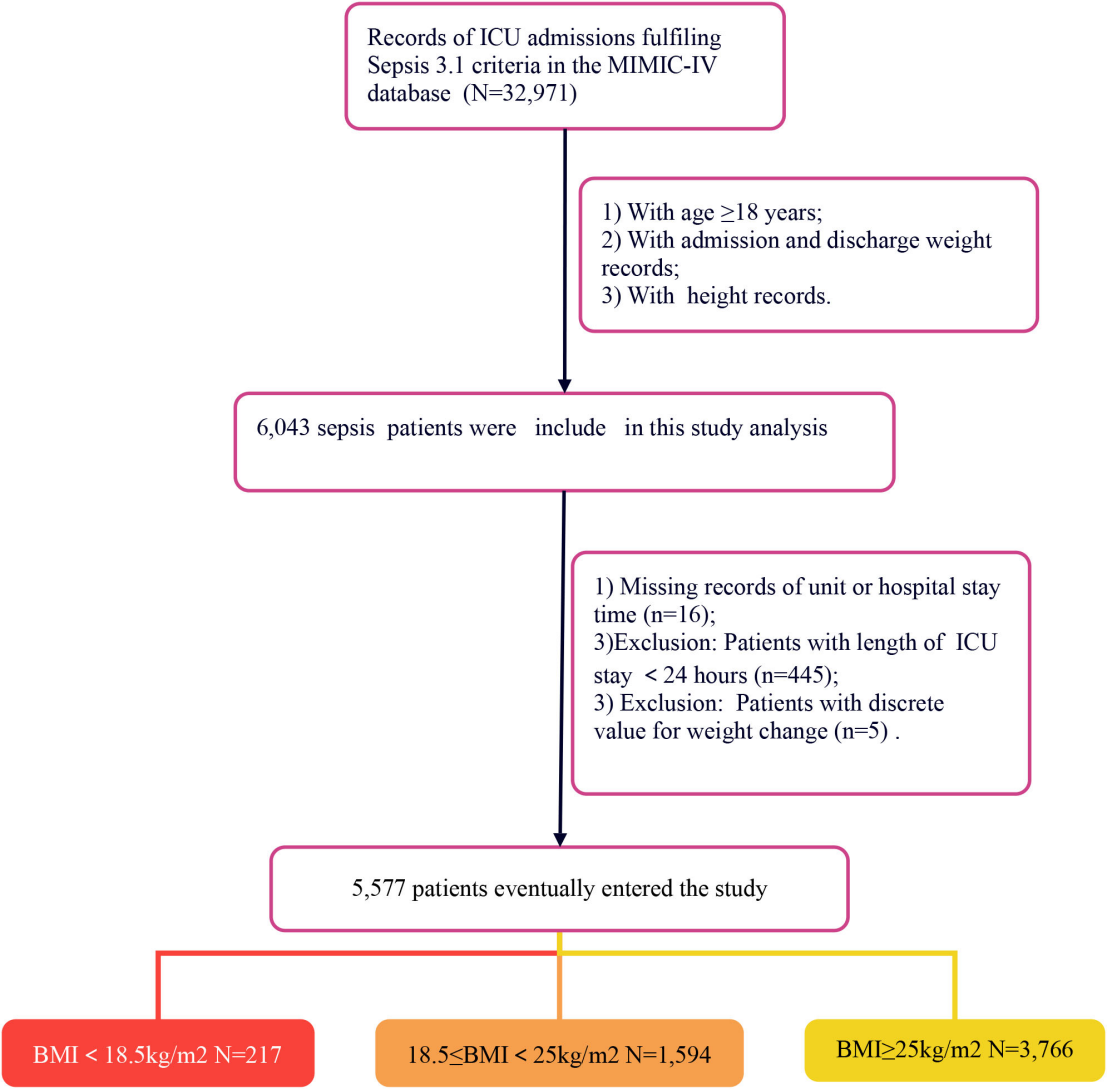
Flowchart of the selection of patients.

Following the process shown in **Figure 1**, 5,577 adult patients with sepsis were ultimately included.

### 2.3 Variable extraction

Using PostgreSQL 17.7 and Navicat Premium 16, SQL queries were executed to extract the following variables: Demographics and vital signs: age, sex, ethnicity, height, admission and discharge weights, BMI, mean heart rate, systolic blood pressure, respiratory rate, temperature, and SpO_2_; Laboratory measures: first values within 24 h of ICU admission for Scr, BUN, glucose, white blood cell count, platelet count, and albumin; Disease severity scores: SOFA, GCS, and SAPS II; Comorbidities: congestive heart failure, chronic kidney disease, peripheral vascular disease, cerebrovascular disease, rheumatic disease/peptic ulcer disease (RD/PUD);

Past medical history: hypertension, myocardial infarction, cardiac surgery, and diabetes; treatment-related variables: ICU length of stay (ICU LOS).

### 2.4 Predictor and outcomes

BMI change was defined as the difference between discharge and admission BMI. For standardization, the BMI change rate was calculated as: BMI change (%) = [(Discharge BMI – Admission BMI)/Admission BMI] × 100%.

Patients were stratified by admission BMI into three groups:

<18.5 kg/m^2^: underweight.

18.5–25 kg/m^2^: normal.

≥25 kg/m^2^: overweight/obese.

Primary outcome: 30-day mortality.

### 2.5 Missing data and multiple imputation

The proportions of missing values were as follows: HR 12 (0.22%), SBP 14 (0.25%), diastolic blood pressure (DBP) 14 (0.25%), RR 12 (0.22%), temperature 89 (1.60%), SpO_2_ 14 (0.25%), calcium 54 (0.97%), albumin 1,861 (33.37%), BUN 20 (0.36%), Scr 18 (0.32%), platelets 98 (1.76%), red blood cell count (RBC) 116 (2.08%), WBC 113 (2.03%), and glucose 10 (0.18%). To minimize potential bias and maximize data utilization, we applied multiple imputation by chained equations (MICE). Variables entered into the imputation model included BMI, comorbidities, past medical history, GCS, SOFA score, SAPS II, HR, SBP, DBP, RR, temperature, SpO_2_, calcium, albumin, BUN, Scr, platelets, RBC, WBC, and glucose. All imputations were performed under the missing-at-random (MAR) assumption (13). MICE was performed with five imputed datasets, and all subsequent analyses were conducted on one representative imputed dataset, as recommended for large-scale observational studies with low overall missingness.

### 2.6 Statistical analysis

All analyses were performed using the R statistical software package (R Foundation for Statistical Computing; http://www.R-project.org) and Fengrui statistical software version 2.2.0. The BMI change rate (%) was calculated as (discharge BMI − admission BMI)/admission BMI × 100%, and patients were classified into three groups: group 1 (decrease in BMI), group 2 (stable BMI), and group 3 (increase in BMI).

Normality of continuous variables was assessed with the Shapiro–Wilk test. Normally distributed data are expressed as mean ± standard deviation (SD) and compared with Student’s t-test or one-way ANOVA. Non-normally distributed data are presented as median [interquartile range (IQR)] and compared with the Wilcoxon rank-sum test. Categorical variables are reported as n (%) and compared using the χ² or Fisher’s exact test.

Multicollinearity was evaluated using the variance inflation factor (VIF); variables with VIF >5 were excluded from multivariable models (14). Kaplan–Meier (KM) curves were used to estimate cumulative all-cause mortality. Three sequential Cox proportional hazards models were constructed: Model 1, unadjusted; Model 2, adjusted for age and sex; and Model 3, adjusted for sex, age, race, ICU length of stay (ICU LOS), congestive heart failure (CHF), renal disease, peripheral vascular disease (PVD), cerebrovascular disease (CVD), heart rate (HR), systolic blood pressure (SBP), respiratory rate (RR), temperature, SpO_2_, albumin, blood urea nitrogen (BUN), serum creatinine (Scr), SOFA, GCS, SAPS II, platelets, red blood cell count (RBC), cardiac surgery, hypertension, and myocardial infarction (MI). Covariates were selected based on clinical relevance and univariate results, ensuring VIF <5 throughout.

Because the BMI change rate is continuous and may have a nonlinear association with mortality, restricted cubic spline (RCS) analysis was used to examine the shape of the relationship between BMI change rate and 30-day mortality in patients with sepsis. To quantify any threshold effect, a two-piecewise linear regression model was applied. The cut-off point for the BMI change rate was determined by maximizing model likelihood while shifting the knot across a predefined range in exploratory analyses. The likelihood ratio test compared the fit of the single linear model with that of the two-piecewise model.

All analyses followed the STROBE guidelines. R software, EmpowerStats, and Fengrui Stats were used; a two-sided *p* < 0.05 was considered statistically significant.

## 3 Results

### 3.1 Characteristics of participants

As shown in **Table 1**, a total of 5,577 ICU patients with sepsis were enrolled. The mean age was 66.5 ± 15.8 years, 57.4% were male, and 2,068 (37.08%) died within 30 days. BMI change ranged from −39% to +49%, with a mean of 0.85% (**Supplementary Figure S1**). After grouping by BMI trajectory during ICU stay, baseline demographics and comorbidities differed markedly across the three groups.

**Table 1:** Baseline characteristics.

Characteristics	All no. (%)	Decrease in BMI	Stable BMI	Increase in BMI	P-value
N	5577	1646	1883	2048	
Age (years)	66.45 ± 15.75	64.41 ± 15.20	67.20 ± 16.29	67.41 ± 15.54	<0.001
LOS-ICU (day)	5.8 (2.8-11.7)	11.19 (6.12-19.85)	2.73 (1.73-4.88)	6.66 (3.76-11.40)	<0.001
Height (cm)	168.78 ± 10.80	169.41 ± 10.55	168.66 ± 10.97	168.38 ± 10.83	<0.001
Admission weight (kg)	83.95 ± 26.93	90.70 ± 27.76	83.08 ± 27.84	79.32 ± 24.16	<0.001
Discharge weight (kg)	84.54 ± 26.43	83.98 ± 25.71	83.09 ± 27.86	86.31 ± 25.55	<0.001
Admission BMI (kg/m2)	29.50 ± 8.71	31.62 ± 8.92	29.15 ± 9.03	28.13 ± 7.89	<0.001
Discharge BMI (kg/m2)	29.60 ± 8.59	29.18 ± 8.22	29.15 ± 9.03	30.37 ± 8.41	<0.001
BMI change rate (%)	0.85 ± 8.75	-7.56 ± 6.19	0.00 ± 0.00	8.38 ± 7.98	<0.001
HR (bpm)	91.61 ± 17.54	91.15 ± 18.04	91.31 ± 17.20	92.26 ± 17.42	<0.001
SBP (mmHg)	110.67 ± 13.45	111.95 ± 14.15	110.61 ± 13.31	109.70 ± 12.90	<0.001
DBP (mmHg)	60.15 ± 9.64	60.65 ± 9.81	60.31 ± 9.71	59.61 ± 9.41	<0.001
RR (bpm)	21.40 ± 4.52	21.85 ± 4.56	21.14 ± 4.50	21.28 ± 4.49	<0.001
T (°C)	36.96 ± 0.66	37.02 ± 0.66	36.90 ± 0.68	36.95 ± 0.63	<0.001
SPO2 (%)	96.60 ± 2.63	96.45 ± 2.48	96.47 ± 2.85	96.85 ± 2.51	<0.001
Gender, n (%)					<0.001
Man	16005 (57.40%)	5135 (62.39%)	5215 (55.39%)	5655 (55.22%)	
Female	11880 (42.60%)	3095 (37.61%)	4200 (44.61%)	4585 (44.78%)	
RACE, n (%)					<0.001
White	18800 (67.42)	5395 (65.55)	6630 (70.42)	6775 (66.16)	
Black	2845 (10.20)	755 (9.17)	1005 (10.67)	1085 (10.60)	
Yellow	955 (3.42)	265 (3.22)	290 (3.08)	400 (3.91)	
Unknown	5285 (18.95)	1815 (22.05)	1490 (15.83)	1980 (19.34)	
Comorbidities, n (%)					
CHF, n (%)	10240 (36.72)	3475 (42.22)	3110 (33.03)	3655 (35.69)	<0.001
PVD, n (%)	3905 (14.00)	1345 (16.34)	1135 (12.06)	1425 (13.92)	<0.001
CVD, n (%)	3905 (14.00)	1345 (16.34)	1135 (12.06)	1425 (13.92)	<0.001
RD, n (%)	1135 (4.07)	345 (4.19)	370 (3.93)	420 (4.10)	0.666
PUD, n (%)	1265 (4.54)	340 (4.13)	330 (3.51)	595 (5.81)	<0.001
Renal Disease, n (%)	7790 (27.94)	2490 (30.26)	2560 (27.19)	2740 (26.76)	<0.001
History, n (%)					
Cardiac surgery, n (%)	6725 (24.12)	2470 (30.01)	1875 (19.92)	2380 (23.24)	<0.001
Diabetes, n (%)	10690 (38.34)	3410 (41.43)	3520 (37.39)	3760 (36.72)	<0.001
Hypertension, n (%)	15980 (57.31)	4680 (56.87)	5330 (56.61)	5970 (58.30)	0.036
MI, n (%)	5690 (20.41)	1835 (22.30)	1760 (18.69)	2095 (20.46)	<0.001
30-day mortality, n (%)	10340 (37.08)	2305 (28.01)	3635 (38.61)	4400 (42.97)	<0.001

Continuous variables are summarized as mean (SD) or median (trisection interval); categorical variables are presented as percentages (%). BMI, body mass index; LOS, length of stay; HR, heart rate; SBP, systolic blood pressure; DB, diastolic blood pressure; RR, respiratory rate; T, Temperature; SPO2, peripheral oxygen saturation; CHF, chronic heart failure; PVD, peripheral vascular disease; CVD, cerebrovascular disease; RD, rheumatic disease; PUD, peptic ulcer disease; MI, myocardial infarction; ICU, intensive care unit.

The BMI-decrease group (n = 1,646) had the highest admission BMI (31.62 kg/m²), an average BMI decline of 7.56%, the longest median ICU stay (11.19 days), and the lowest 30-day mortality (28.01%). The BMI-stable group (n = 1,883) appeared least ill, with the highest median GCS (13 points) and the lowest SOFA and SAPS II scores, and a 30-day mortality of 38.61%. The BMI-increase group (n = 2,048) had the lowest admission BMI (28.13 kg/m²), gained 8.4% in weight, and showed the highest 30-day mortality (42.97%). Significant differences were also observed among the groups in sex, ethnicity, comorbidities (heart failure, peripheral vascular disease, chronic kidney disease, etc.), vital signs, and ICU length of stay (all *p* < 0.05). Detailed laboratory findings are reported in **Table 2** and the **Supplementary Results**.

**Table 2:** Laboratory parameters and severity scores.

Characteristics	All no. (%)	Decrease in BMI	Stable BMI	Increase in BMI	P-value
N	5577	1646	1883	2048	
Calcium (mg/dL)	7.73 ± 0.95	7.79 ± 0.96	7.73 ± 0.90	7.69 ± 0.97	<0.001
ALB	2.70 ± 0.88	2.74 ± 0.89	2.70 ± 0.87	2.68 ± 0.87	<0.001
BUN (mg/dL)	26.00 (15.00-43.00)	26.00 (16.00-45.00)	26.00 (15.00-43.00)	26.00 (15.00-42.00)	0.023
Scr (mg/dL)	1.20 (0.80-2.10)	1.20 (0.80-2.20)	1.20 (0.80-2.00)	1.20 (0.80-2.00)	<0.001
GCS score	9.00 (5.00-14.00)	8.00 (3.00-14.00)	13.00 (7.00-15.00)	9.00 (5.00-14.00)	<0.001
SOFA score	7.98 ± 4.07	8.33 ± 4.01	7.50 ± 4.16	8.14 ± 3.99	<0.001
SAPSII score	46.84 ± 15.86	46.71 ± 15.27	45.34 ± 16.89	48.34 ± 15.21	<0.001
PTL (10^9^/L)	212.06 ± 129.44	208.60 ± 121.10	212.27 ± 131.52	214.64 ± 133.86	0.007
RBC (10^12^/L)	3.54 ± 0.81	3.59 ± 0.85	3.50 ± 0.79	3.54 ± 0.81	<0.001
WBC (109/L)	14.53 ± 11.97	13.90 ± 10.01	14.72 ± 11.62	14.87 ± 13.59	<0.001
Glucose (mg/dL)	158.00 ± 108.99	161.59 ± 103.82	156.26 ± 109.09	156.71 ± 112.84	0.002

Continuous variables are summarized as mean (SD) or median (trisection interval); ALB, albumin; BUN, blood urea nitrogen; Scr, serum creatinine; SOFA, sequential organ failure assessment; GCS, Glasgow coma scale; SAPS II score, simplified acute physiology score I; PTL, platelet count; RBC, red blood cell count; WBC, white blood cell count.

### 3.2 Kaplan–Meier analysis of BMI change and 30-day mortality in ICU sepsis patients

Overall Kaplan–Meier curves showed that the BMI-decrease group had markedly lower 30-day mortality than the stable or increase groups (log-rank *p* < 0.001). Subgroup analyses confirmed this benefit across all baseline BMI strata: underweight (<18.5 kg/m²), normal weight (18.5–24.9 kg/m²), and overweight/obese (≥25 kg/m²) (all subgroups log-rank *p* < 0.01) (**Supplementary Figure S2**).

### 3.3 Factors affecting the risk of 30-day mortality were examined using univariate Cox proportional hazards regression analysis

Univariate Cox analysis (**Supplementary Table S1**) showed that each one-year increase in age raised the risk of death by 2% (hazard ratio [HR] 1.02; *p* < 0.001), whereas every 1% decrease in BMI increased the risk by 3% (HR 1.03; *p* < 0.001). Admission BMI was inversely associated with mortality (HR 0.99; *p* < 0.001). Race unknown, congestive heart failure (CHF), peripheral vascular disease (PVD), cerebrovascular disease (CVD), renal disease, low systolic blood pressure (SBP), low SpO_2_, low albumin, high respiratory rate (RR), high SOFA and SAPS II scores, low Glasgow Coma Scale (GCS) score, and low red blood cell count (RBC) were all significantly associated with increased 30-day mortality (all *p* < 0.001). Sex, Black race, rheumatic disease (RD), peptic ulcer disease (PUD), diabetes, blood glucose, and calcium levels showed no significant association with death.

### 3.4 Results from multivariate analyses using Cox proportional hazards regression methods

**Table 3** summarizes the results of the multivariable Cox proportional hazards regression models, which systematically characterize the relationship between BMI change rate and 30-day mortality in ICU sepsis patients. After full adjustment, each 1% increase in BMI change (i.e., less decline or actual gain) was independently associated with higher 30-day mortality (HR 1.02; 95% confidence interval [CI] 1.02–1.02; *p* < 0.001).

**Table 3:** Associations between BMI change rate and risk of 30-day mortality.

Group	Exposure	Number	Event (%)	Model I (HR,95%CI) P	Model II (HR,95%CI) P	Model III (HR,95%CI) P
All (N = 5577)	BMI change	5577	2068 (37.08)	1.03 (1.02, 1.03) <0.0001	1.03 (1.02, 1.03) <0.0001	1.02 (1.02, 1.02) <0.0001
	Decrease in BMI	1646	461 (28.01)	1(Ref)	1(Ref)	1(Ref)
	Stable BMI	1883	727 (38.61)	1.62 (1.54, 1.71) <0.0001	1.55 (1.47, 1.64) <0.0001	1.56 (1.47, 1.66) <0.0001
	Increase in BMI	2048	880 (42.97)	1.72 (1.64, 1.81) <0.0001	1.64 (1.56, 1.73) <0.0001	1.58(1.48, 1.64) <0.0001
BMI<18.5kg/m2 (N = 217)	BMI change	217	89 (41.94)	1.01 (1.00, 1.02) 0.0130	1.01 (1.00, 1.02) 0.0128	0.99 (0.98, 1.00) 0.0930
	Decrease in BMI	29	9 (31.03)	1(Ref)	1(Ref)	1(Ref)
	Stable BMI	81	33 (40.74)	1.59 (1.14, 2.21) 0.0059	1.32 (0.94, 1.84) 0.1080	1.03 (0.71, 1.49) 0.8654
	Increase in BMI	107	49 (45.79)	1.65 (1.20, 2.27) 0.0020	1.41 (1.02, 1.94) 0.0369	1.03 (0.72, 1.46) 0.8811
18.5kg/m2≤BMI<25kg/m2 (N = 1594)	BMI change	1594	624 (39.15)	1.02 (1.02, 1.02) <0.0001	1.02 (1.02, 1.02) <0.0001	1.02 (1.01, 1.02) <0.0001
	Decrease in BMI	333	106 (31.83)	1(Ref)	1(Ref)	1(Ref)
	Stable BMI	568	220 (38.73)	1.41 (1.27, 1.56) <0.0001	1.34 (1.21, 1.49) <0.0001	1.30 (1.16, 1.45) <0.0001
	Increase in BMI	693	298 (43.00)	1.49 (1.35, 1.65) <0.0001	1.43 (1.30, 1.58) <0.0001	1.27 (1.15, 1.41) <0.0001
BMI≥25kg/m2 (N = 3766)	BMI change	3766	1353 (35.93)	1.03 (1.03, 1.04) <0.0001	1.03 (1.03, 1.03) <0.0001	1.03 (1.03, 1.03) <0.0001
	Decrease in BMI	1284	346 (26.95)	1(Ref)	1(Ref)	1(Ref)
	Stable BMI	1234	474 (38.41)	1.69 (1.59, 1.79) <0.0001	1.63 (1.53, 1.74) <0.0001	1.68 (1.57, 1.81) <0.0001
	Increase in BMI	1248	533 (42.71)	1.81 (1.70, 1.92) <0.0001	1.72 (1.62, 1.83) <0.0001	1.72 (1.61, 1.84) <0.0001

Model I: we did not account for additional variables.

Model II: we adjusted gender, age, and race.

Model III: we adjusted gender, age, race, LOS-ICU, CHF, renal disease, PVD; CVD; HR; SBP; RR; T; SPO2; ALB; BUN; creatinine; SOFA; GCS; SAPSII; PLT; RBC; cardiac surgery; hypertension; MI. HR, hazard ratios; CI, confidence; Ref, reference.

When patients were divided into three trajectory groups, the BMI-stable group showed a markedly higher risk of death compared with the BMI-decrease group (HR 1.56; 95% CI 1.47–1.66; *p* < 0.001), whereas the BMI-increase group bore the greatest risk (HR 1.58; 95% CI 1.48–1.64; *p* < 0.001). Stratified analyses revealed that the association between BMI change and 30-day mortality was not uniform across baseline BMI categories.

In patients with BMI <18.5 kg/m², BMI change rate was not significantly related to mortality (HR 0.99; 95% CI 0.98–1.00; *p* = 0.093). Among those with BMI 18.5–24.9 kg/m², each 1% increase in BMI change was associated with a 2% rise in mortality risk (HR 1.02; 95% CI 1.01–1.02; *p* < 0.001); compared with the BMI-decrease group, both the BMI-stable (HR 1.30; 95% CI 1.16–1.45; *p* < 0.001) and BMI-increase (HR 1.27; 95% CI 1.15–1.41; *p* < 0.001) groups exhibited significantly higher 30-day mortality.

In patients with BMI ≥25 kg/m², the same positive association was observed (HR 1.03 per 1% increase; 95% CI 1.03–1.03; *p* < 0.001). Relative to the BMI-decrease group, the BMI-stable group had an HR of 1.68 (95% CI 1.57–1.81; *p* < 0.001) and the BMI-increase group an HR of 1.72 (95% CI 1.61–1.84; *p* < 0.001).

### 3.5 Sensitivity analysis

Using the original non-imputed data (n = 5,577; albumin 33% missing), each 1% increase in BMI change rate remained significantly associated with higher 30-day mortality (hazard ratio [HR] 1.02; 95% confidence interval [CI] 1.01–1.02; *p* < 0.0001). Trajectory-specific hazard ratios differed by <5% from the imputed analysis and retained the same direction and statistical significance (**Supplementary Table S3**). Thus, our conclusions are not dependent on the multiple imputation procedure.

### 3.6 Nonlinear relationship assessed with restricted cubic splines in Cox regression

**Figure 2:**
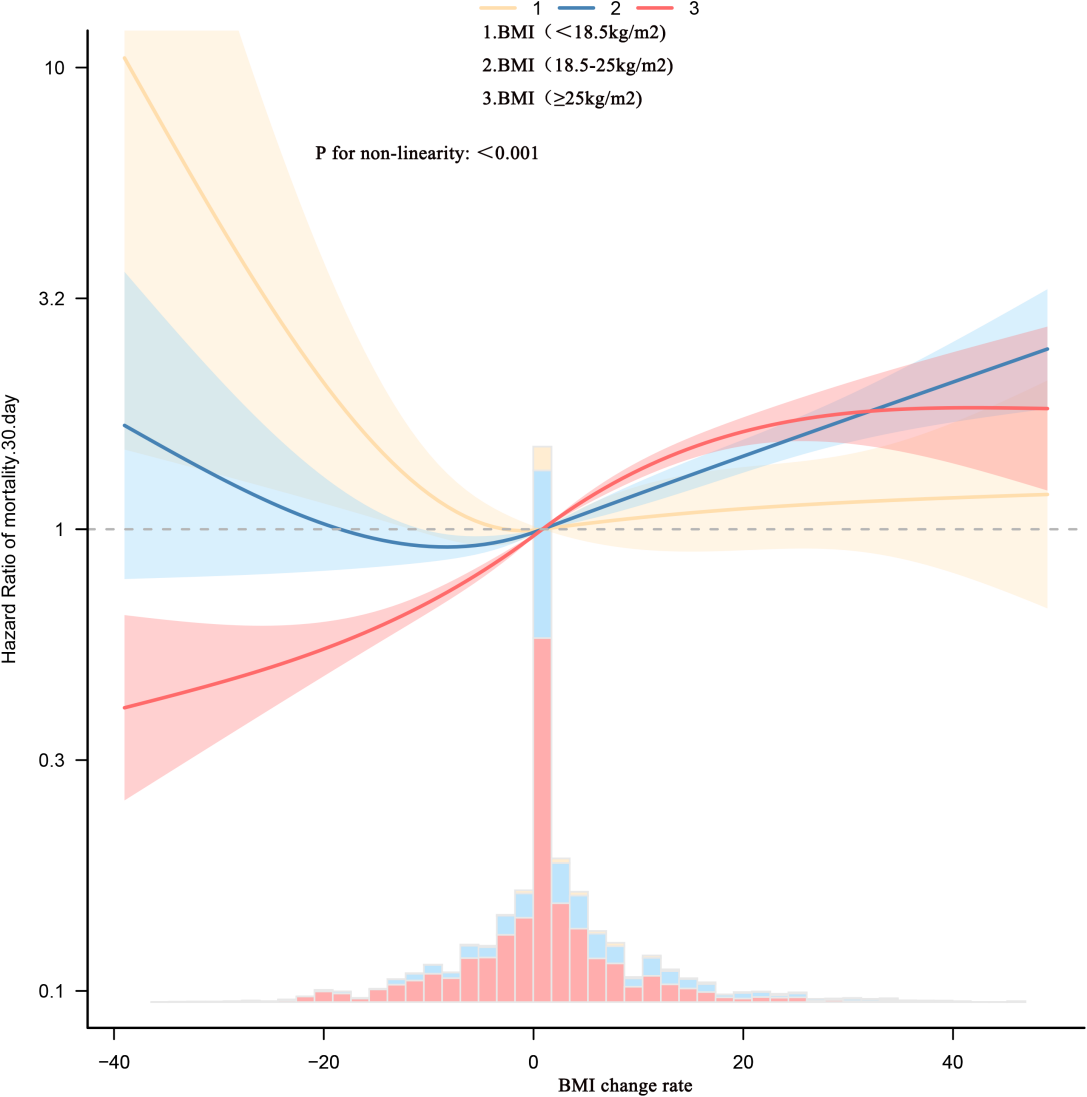
Dose-response association between absolute BMI change rate across adulthood and risk of all-cause mortality. Associations were examined using multivariable Cox regression models based on restricted cubic splines. The solid line represents estimated hazard ratios and the dashed line represents 95% CIs. Risk estimates were adjusted for baseline gender, age, race, LOS-ICU, CHF, renal disease, PVD; CVD; HR; SBP; RR; T; SPO2; ALB; BUN; creatinine; SOFA; GCS; SAPSII; PLT; RBC; cardiac surgery; hypertension; MI. These thresholds are exploratory and should not be used as treatment targets without prospective validation.

To explore the nonlinear association between BMI change rate and 30-day mortality in patients with sepsis, Cox models with restricted cubic splines were fitted. After stratifying patients by admission BMI, **Figure 2** and **Table 4** show that BMI change displayed nonlinear relationships with mortality in every stratum, each with a distinct inflection point. Admission BMI < 18.5 kg/m² – Inflection point: –2%. To the left of –2%, each 1% increase in BMI (i.e., less decline) reduced the risk of death by 5% (HR 0.95, 95% CI 0.96–0.97). To the right of –2%, BMI change was no longer significantly associated with mortality (HR 1.00, 95% CI 0.99–1.01).

**Table 4:** Threshold effect analysis of BMI change and 30-day mortality.

Outcome: risk of mortality	BMI <18.5kg/m^2^ HR, 95%CI P -value	BMI (18.5-25kg/m^2^) HR, 95%CI P -value	BMI ≥25kg/m^2^ HR, 95%CI P -value
Model I	0.99 (0.98, 1.00) 0.0930	1.02 (1.01, 1.02) <0.0001	1.03 (1.03, 1.03) <0.0001
Model II			
Inflection points of BMI change (%)	-2	4	-1
<K	0.95 (0.92, 0.99) 0.0124	1.00 (0.99, 1.01) 0.5026	1.04 (1.03, 1.05) <0.0001
≥K	1.00 (0.99, 1.01) 0.4809	1.03 (1.02, 1.03) <0.0001	1.02 (1.02, 1.03) <0.0001
P for log-likelihood ratio test	1.05 (1.00, 1.09) 0.0314	1.03 (1.02, 1.04) <0.0001	0.98 (0.98, 0.99) 0.0013
	0.038	<0.001	0.001

Data were presented as HR (95% CI) P value; Model I, linear analysis; Model II, non-linear analysis. Adjusted for gender, age, race, LOS-ICU, CHF, renal disease, PVD; CVD; HR; SBP; RR; T; SPO2; ALB; BUN; creatinine; SOFA; GCS; SAPSII; PLT; RBC; cardiac surgery; Hypertension; MI. K indicates inflection point. *P < 0.05 indicates that model II is significantly different from Model I.

18.5 ≤ Admission BMI < 25 kg/m² Inflection point: +4%. Left of +4%, BMI change was not significantly related to mortality (HR 1.00, 95% CI 0.99–1.01).

– Right of +4%, each 1% increase in BMI raised the risk by 3% (HR 1.03, 95% CI 1.02–1.03). Admission BMI ≥ 25 kg/m² Inflection point: –1%. Regardless of the side of the inflection point, mortality risk increased with rising BMI. Notably, the magnitude of increase was larger to the left of –1%: each 1% increase in BMI conferred a 4% higher risk (HR 1.04, 95% CI 1.03–1.05), whereas to the right the risk rose by 2% per 1% increase (HR 1.02, 95% CI 1.02–1.03).

### 3.7 Predictive value of dynamic BMI change for 30-day mortality in ICU sepsis patients

Both the absolute BMI change and the BMI change rate demonstrated the highest discriminatory ability for 30-day mortality (area under the curve [AUC] 0.5828; 95% CI 0.576–0.5895 and AUC 0.584; 95% CI 0.5771–0.5908, respectively). These values were significantly superior to those of admission or discharge weight and BMI (AUC 0.5032–0.5391; all *p* < 0.001) (see **Supplementary Figure S3**, **Supplementary Table S4**).

## 4 Discussion

This study examined the association between dynamic changes in BMI during ICU stay and 30-day mortality in patients with sepsis, stratifying analyses by baseline BMI. Four principal findings emerged. A direct, linear relationship was observed: after full adjustment, every 1% increase in BMI change (relative to admission) was associated with a 2% rise in 30-day mortality (adjusted HR 1.02; 95% CI 1.02–1.02; *p* < 0.0001). Paradoxically, patients whose BMI declined exhibited a survival advantage, recording the lowest 30-day mortality (28.0%) despite presenting with the highest admission BMI. Stratification by baseline BMI revealed heterogeneity. Among patients with normal or elevated BMI (≥18.5 kg/m²), BMI change remained positively correlated with mortality. In contrast, among underweight patients (<18.5 kg/m²), BMI change was not significantly related to death risk. Restricted cubic spline analyses disclosed nonlinear relationships whose inflection points differed across BMI strata: −2% for BMI <18.5 kg/m², +4% for 18.5–24.9 kg/m², and −1% for ≥25 kg/m². Incorporating BMI change into predictive models yielded better discrimination for 30-day mortality than admission BMI alone (combined AUC 0.58 vs. 0.53).

Previous studies have demonstrated a complex relationship between BMI and mortality in critically ill patients. Some investigations indicate that elevated BMI—especially obesity—significantly increases short-term mortality among ICU or sepsis patients (15, 16). Conversely, a landmark meta-analysis conducted by Bai et al., encompassing 105,159 sepsis patients, revealed a classic U-shaped association: overweight (BMI 25–29.9 kg/m²) and obese (BMI 30–39.9 kg/m²) individuals exhibited 21% and 26% reductions in death risk, respectively, whereas underweight patients (BMI <18.5 kg/m²) faced a 2.42-fold increase in long-term mortality (HR 2.42; 95% CI 2.07–2.84) (4, 5). This “obesity paradox” suggests that adipose tissue may provide metabolic reserves and exert anti-inflammatory protection during acute stress states (17). Our study further elucidates this paradox by demonstrating that the protective association of higher baseline BMI is contingent upon subsequent weight dynamics. Specifically, we observed that obese patients exhibiting a moderate BMI reduction experienced the lowest mortality risk, whereas those with significant weight gain faced increased fatality. This suggests that the obesity paradox in sepsis may not solely relate to static adiposity but also to the metabolic adaptability and fluid balance reflected in BMI trajectories during critical illness.

During the 30-day follow-up, we documented 2,068 deaths among ICU patients with sepsis, corresponding to a mortality rate of 37.08%—a figure in line with previous reports from European and North American cohorts (18–20). Notably, patients presenting with higher admission BMI were more likely to experience BMI decline yet exhibited the lowest 30-day mortality (28.01%). Conversely, those with the lowest admission BMI, despite an 8.4% weight gain, still had the highest overall mortality (42.97%). Our findings thus corroborate the widely observed “obesity paradox.”

However, mounting evidence questions the clinical utility of static BMI measurements. Zhang et al. analyzed 299,712 ICU patients and found that every 1% increase in BMI during hospitalization was associated with a 36% higher ICU mortality risk (OR 1.36; *p* < 0.001) (10). Similarly, another study showed that a >4% BMI rise doubled the risk of death. In patients on continuous ambulatory peritoneal dialysis (CAPD), greater BMI loss within the first year was linked to increased all-cause mortality independent of baseline BMI (21), while an unintentional BMI decline in Parkinson’s disease tripled the risk of death (22). Nevertheless, none of these investigations focused specifically on sepsis, and no study has yet quantified the impact of BMI change—measured from ICU admission to discharge—on mortality across strata of baseline BMI. In the setting of sepsis, where large volume shifts and hypercatabolism are common, it remains unclear whether weight fluctuations are merely passive markers of disease severity or modifiable determinants of outcome.

Therefore, the present study investigated the relationship between inpatient BMI change and short-term outcomes in ICU patients with sepsis. First, multivariable Cox regression revealed that, after full adjustment for confounders, the rate of BMI change remained independently and positively associated with 30-day mortality—findings that align with the recent report by Zhang et al. (10). Their study included all ICU diagnoses, thus mixing trauma, postoperative states, acute pancreatitis, and other conditions; in contrast, we strictly applied the Sepsis 3.0 criteria (SOFA ≥2) to ensure our conclusions are directly relevant to evidence-based sepsis care. Moreover, we leveraged the high-quality, single-center MIMIC-IV dataset spanning 2008–2022, which offers longer follow-up and lower center heterogeneity than the 2014–2015 eICU-CRD cohort used by Zhang et al. The concordant result—BMI gain increases death risk—enhances the robustness and generalizability of the finding. A further distinction lies in our stratified analyses. After classifying patients by admission BMI, we observed that BMI change rate was not significantly related to 30-day mortality in the underweight stratum (<18.5 kg/m²), whereas a positive relationship persisted among patients with normal or elevated BMI (≥18.5 kg/m²). These data suggest that underweight sepsis patients may benefit from targeted weight gain, whereas those with higher baseline BMI may require controlled weight reduction to mitigate mortality.

Importantly, restricted cubic spline analyses revealed a nonlinear relationship between BMI change rate and 30-day mortality across all admission BMI strata, each characterized by a distinct inflection point. Among patients with admission BMI <18.5 kg/m², the inflection point was −2%. Left of −2%, each 1% increase in BMI (i.e., less decline) was associated with a 5% reduction in death risk (HR 0.95). Right of −2%, BMI change was no longer significantly related to mortality, implying that permitting a modest 2% BMI decline in this underweight cohort reflects successful fluid de-resuscitation without additional survival benefit from further loss or gain. For patients with admission BMI 18.5–24.9 kg/m², the inflection point was +4%. Left of +4%, BMI changes did not materially affect mortality. Once BMI rose beyond +4%, every 1% increase was linked to a 3% rise in death risk (HR 1.03). This suggests a ceiling of 4% BMI loss; any subsequent gain should be avoided through strict fluid balance and prevention of overnutrition. In patients with admission BMI ≥25 kg/m², the inflection point was −1%. On either side of −1%, mortality increased with BMI gain, but the slope was steeper left of −1% (HR 1.04 per 1% increase) than right of −1% (HR 1.02). Thus, targeting approximately a 1% BMI reduction appears optimal for obese sepsis patients, warranting deliberate negative fluid balance or moderate caloric restriction. Traditional “one-size-fits-all” weight management neglects baseline nutritional status. These data indicate that ICU teams can set patient-specific BMI change limits immediately upon admission, thereby transforming the simple “scale” into a real-time therapeutic monitor.

Furthermore, our study confirmed that the discriminative power of BMI change rate (AUC 58.40%) for predicting 30-day mortality in sepsis was superior to that of a single admission BMI measurement (AUC 53.48%). These findings are consistent with multicenter cohort data: Zhang et al. (10) reported that BMI change rate yielded a significantly higher AUC for ICU mortality than baseline BMI, and Shimizu et al. (23) demonstrated that incorporating BMI change velocity improved the prediction of sepsis mortality from an AUC of 0.71 (baseline BMI model) to 0.79.

The mechanisms underlying our observations may be best explained by fluid shifts rather than changes in lean body mass. In the critical phase of sepsis, the initial BMI decrease observed in our cohort—particularly among those with higher admission BMI—likely reflects successful fluid mobilization and de-resuscitation following the resolution of the systemic inflammatory response and capillary leakage, rather than catastrophic muscle wasting (24, 25). This negative fluid balance is associated with improved organ function and survival. Conversely, a positive BMI change may signal persistent capillary leakage, fluid overload, or potential overfeeding, all of which are established drivers of multiorgan dysfunction and worse outcomes (26, 27). This interpretation is further supported by the differential effects across BMI strata: in obese patients, the relative stability or mild reduction in BMI, coupled with potential metabolic and anti-inflammatory reserves in adipose tissue, may confer a survival advantage (the “obesity paradox”) (17). In stark contrast, underweight patients possess minimal metabolic and physiological reserves. Consequently, any further reduction in BMI—even if partly fluid—may push them beyond a critical threshold, unmasking their vulnerability and explaining the lack of benefit from weight loss, a phenomenon analogous to the “low-BMI mortality paradox” seen in other chronic conditions (28).

Our study demonstrates a nonlinear, BMI stratum–specific relationship between BMI change rate and 30-day mortality in ICU patients with sepsis. At the public health level, these findings support integrating dynamic weight monitoring into routine surveillance to inform precise nutrition and fluid management policies. Clinically, they shift the paradigm from a single static BMI assessment to continuous tracking, enabling clinicians to set individualized weight-safety thresholds based on identified inflection points. This facilitates early risk re-stratification, guides fluid balance and nutritional interventions, and ultimately helps reduce short-term sepsis mortality.

Our study has several notable strengths. First, we leveraged the MIMIC-IV database (2008–2022) to assemble a large cohort of 5,577 ICU patients with sepsis who strictly fulfilled the Sepsis 3.0 criteria; the substantial sample size and complete follow-up endow the analyses with high statistical power. Second, we performed, to our knowledge, the first full-trajectory dynamic assessment of body mass index (BMI) change by quantifying the rate of change across the entire ICU stay—from admission to discharge—and used restricted cubic splines to identify inflection points within each baseline BMI stratum, yielding clinically actionable thresholds for precision weight management. Third, we stratified patients into three baseline BMI groups (<18.5, 18.5–24.9, and ≥25 kg/m²) and applied two-piecewise linear regression to confirm nonlinear relationships, thereby avoiding the “one-size-fits-all” bias inherent to static BMI measurements. Fourth, we rigorously addressed missing data: key variables with up to 33% missingness (e.g., albumin) were multiply imputed via chained equations, and multivariable models adjusted for more than 20 covariates while maintaining variance inflation factors below 5, markedly reducing residual confounding. Finally, the BMI change rate demonstrated superior predictive performance compared with admission BMI alone, providing incremental value for early risk re-stratification in sepsis.

Several limitations should be acknowledged. First, although the cohort is large, all data originate from a single tertiary care ICU in the United States, so external validity remains to be confirmed in multicenter databases such as eICU-CRD or JIPAD. Second, BMI change derived from serial weight recordings cannot differentiate among alterations in fat, muscle, or water, rendering the metric vulnerable to bias from fluid resuscitation, diuretics, or edema. Third, the study endpoint was limited to 30-day all-cause mortality; ICU-acquired functional impairment, longer-term survival (90 days or 1 year), and readmission rates were not examined, precluding a comprehensive assessment of the long-term impact of BMI management. Fourth, despite adherence to STROBE guidelines, the retrospective design precludes causal inference, and prospective interventional trials are still required to validate the safety and efficacy of BMI-targeted strategies. Finally, the retrospective nature of MIMIC-IV precludes capture of daily medication (e.g., corticosteroids, vasopressors, diuretics), nutritional prescriptions, and granular intake–output charts. These factors can acutely alter weight independent of tissue mass, potentially biasing the observed associations. Prospective studies with high-resolution drug and fluid balance data are warranted to validate our exploratory thresholds.

## 5 Conclusion

Longitudinal analysis of 5,577 patients with sepsis from MIMIC-IV showed that every 1% rise in ICU BMI increased 30-day mortality by 2%. Exploratory, BMI-specific inflection points occurred at −2% for underweight, +4% for normal weight, and −1% for overweight/obese patients; these thresholds await prospective validation. Dynamic BMI trajectories predicted outcomes better than admission BMI alone. By translating the “obesity paradox” into a bedside instrument, clinicians can (i) set individualized BMI change limits anchored to baseline BMI, (ii) embed daily weight tracking in sepsis quality metrics, (iii) trigger early risk re-stratification, and (iv) fine-tune fluid and nutrition therapy to reduce short-term mortality.

## Data Availability

The datasets presented in this study can be found in online repositories. The names of the repository/repositories and accession number(s) can be found in the article/**Supplementary Material**.
