# Correction: Association of body mass index changes with short-term mortality risks in ICU patients with sepsis across different admission BMI states: analysis of the MIMIC-IV database

**DOI:** 10.3389/fimmu.2026.1801573

**Published:** 2026-02-23

**Authors:** Wei Liu, Wenfei Zeng, Zhenhua Huang, Qinghua Yuan

**Affiliations:** 1 Department of Emergency Medicine, The Huangpu People’s Hospital of, Zhongshan, Zhongshan, China; 2 Department of Anesthesiology, Hunan Provincial People’s Hospital, The First Affiliated Hospital of Hunan Normal University, Changsha, China; 3 Department of Emergency Medicine, the First Affiliated Hospital of Shenzhen University, 7Shenzhen Second People’s Hospital, Shenzhen, China; 4 Department of Cardiology, the Seventh Affiliated Hospital of Sun Yat-sen University, Shenzhen, Guangdong, China

**Keywords:** sepsis, body-mass-index change, intensive care unit (ICU), 30-day mortality, MIMIC-IV database

The reference for “Assessment of global incidence and mortality of hospital-treated sepsis.” was erroneously written as “Fleischmann C, Scherag A, Adhikari NK, Hartog CS, Tsaganos T, Schlattmann P, et al. Assessment of global incidence and mortality of hospital-treated sepsis. *Am J Respir Crit Care Med*. (2016) 193(3):259-72. doi: 10.1164/rccm.201504-0781OC”. It should be “Fleischmann C, Scherag A, Adhikari NKJ, Hartog CS, Tsaganos T, Schlattmann P, et al. Assessment of Global Incidence and Mortality of Hospital-treated Sepsis. Current Estimates and Limitations. *Am J Respir Crit Care Med*. (2016) 193(3):259-72. doi: 10.1164/rccm.201504-0781OC”.

The reference for “Incidence and mortality of hospital- and ICU-treated sepsis: results of a systematic review and meta-analysis.” was erroneously written as “Fleischmann-Struzek C, Goldfarb DM, Schlattmann P, Scherag A, Reinhart K, Kissoon N. Incidence and mortality of hospital- and ICU-treated sepsis: results of a systematic review and meta-analysis. *Intensive Care Med*. (2020) 46(8):1552-62. doi: 10.1007/s00134-020-06151-x”. It should be “Fleischmann-Struzek C, Mellhammar L, Rose N, Cassini A, Rudd KE, Schlattmann P, et al. Incidence and mortality of hospital- and ICU-treated sepsis: results from an updated and expanded systematic review and meta-analysis. *Intensive Care Med*. (2020) 46(8):1552-62. doi: 10.1007/s00134-020-06151-x”.

The reference for “Association between BMI changes and all-cause mortality in Parkinson’s disease.” was erroneously written as “Yoon SY, Choi JY, Nam GE, Han K, Kim DH, Koo J, et al. Association between BMI changes and all-cause mortality in Parkinson’s disease. *J Parkinsons Dis*. (2024) 14(7):1441-50. doi: 10.3233/JPD-240181”. It should be “Yoon SY, Choi JY, Nam GE, Jung J, Han K, Kang SH, et al. Association Between Body Mass Index Changes and All-Cause Mortality in Parkinson’s Disease. *J Parkinsons Dis*. (2024) 14(7):1441-50. doi: 10.3233/JPD-240181”.

The reference for “Lean body mass equations and sepsis outcomes.” was erroneously written as “Shimizu R, Nakanishi N, Ishihara M, Takahashi Y, Kawazoe Y. Lean body mass equations and sepsis outcomes. *Diseases*. (2024) 12(2):30. doi: 10.3390/diseases12020030”. It should be “Shimizu R, Nakanishi N, Ishihara M, Oto J, Kotani J. Utility of Lean Body Mass Equations and Body Mass Index for Predicting Outcomes in Critically Ill Adults with Sepsis: A Retrospective Study. *Diseases*. (2024) 12(2):30. doi: 10.3390/diseases12020030”.

The reference for “Obesity and ICU outcomes: an international cohort study.” was erroneously written as “Arabi YM, Dara SI, Tamim HM, Rishu AH, Bouchama A, Khedr MK, et al. Obesity and ICU outcomes: an international cohort study. *Intensive Care Med*. (2008) 34(12):1999-2009. doi: 10.1007/s00134-008-1239-2”. It should be “Ziaka M, Exadaktylos A. Fluid management strategies in critically ill patients with ARDS: a narrative review. *Eur J Med Res* (2025) 30(1):401. doi:10.1186/s40001-025-02661-w”.

The reference for “BMI and weight loss on the kidney transplant wait-list.” was erroneously written as “Molnar MZ, Streja E, Kovesdy CP, Bunnapradist S, Sampaio MS, Jing J, et al. BMI and weight loss on the kidney transplant wait-list. *Am J Transplant*. (2011) 11(4):725-36. doi: 10.1111/j.1600-6143.2011.03468.x”. It should be “Molnar MZ, Streja E, Kovesdy CP, Bunnapradist S, Sampaio MS, Jing J, et al. Associations of body mass index and weight loss with mortality in transplant-waitlisted maintenance hemodialysis patients. *Am J Transplant*. (2011) 11(4):725-36. doi: 10.1111/j.1600-6143.2011.03468.x”.

The original version of this article has been updated.

