# Supplementary material for: Association of body mass index changes with short-term mortality risks in ICU patients with sepsis across different admission BMI states: analysis of the MIMIC-IV database
Source: Front Immunol. 2025 Oct 24;16:1698405. doi: 10.3389/fimmu.2025.1698405 (PMC12591966; doi:10.3389/fimmu.2025.1698405)
Supplement: Supplementary file 1 [file DataSheet1.docx]

**Association of** **Body Mass Index (BMI) Changes with Short-term** **Mortality Risks in ICU Patients with Sepsis** **across different admission BMI states: Analysis of the MIMIC-IV database**

**Wei Liu^1†^,** **Wenfei Zeng^2†^, Zhenhua Huang^3*^, Qinghua Yuan^4*^**

1Department of Emergency Medicine, The Huangpu People’s Hospital of Zhongshan, Zhongshan, China

2Department of Anesthesiology, Hunan Provincial People's Hospital, The First Affiliated Hospital of Hunan Normal University, Changsha, 410005, China

3Department of Emergency Medicine, the First Affiliated Hospital of Shenzhen University, 7Shenzhen Second People’s Hospital, Shenzhen, 518035, China.

4Department of Cardiology, the Seventh Affiliated Hospital of Sun Yat-sen University, Shenzhen, 518000, Guangdong, China.

**Supplementary Results**
**Table 2** summarizes the first 24-hour laboratory values and illness-severity scores across BMI-trajectory groups. Compared with the other two trajectories, the BMI-decrease group showed marginally higher calcium and albumin concentrations, together with the highest SOFA and SAPS II scores, indicating greater baseline severity. The BMI-stable group had the most favourable profile, including the highest GCS and the lowest SOFA and SAPS II scores. The BMI-increase group exhibited intermediate biochemical values but the highest median WBC count, while renal-function markers (BUN, Scr) were comparable among the three groups (all *P* < 0.05).

Table S1. Factors influencing risk of mortality analyzed by univariate Cox proportional hazards regression analysis

| Variable | Characteristics | HR (95% CI) P-value |
| --- | --- | --- |
| Gender, n (%) |  |  |
| Man | 16005 (57.40%) | 1.0 |
| Female | 11880 (42.60%) | 1.00 (0.97, 1.04) 0.809 |
| Age (years) | 66.45 ± 15.75 | 1.02 (1.02, 1.02) <0.001 |
| RACE, n (%) |  |  |
| Whight | 18800 (67.42%) | 1.0 |
| Black | 2845 (10.20%) | 0.98 (0.92, 1.05) 0.5200 |
| Yellow | 955 (3.42%) | 1.15 (1.04, 1.28) 0.008 |
| Unknown | 5285 (18.95%) | 1.26 (1.20, 1.32) <0.001 |
| LOS-ICU (day) | 5.8 (2.8-11.7) | 0.97 (0.97, 0.97) <0.001 |
| Admission BMI (kg/m2) | 29.50 ± 8.71 | 0.99 (0.99, 0.99) <0.001 |
| Discharge BMI (kg/m2) | 29.60 ± 8.59 | 1.00 (1.00, 1.00) 0.341 |
| BMI change rate (%) | 0.85 ± 8.75 | 1.03 (1.02, 1.03) <0.001 |
| CHF, n (%) | 10240 (36.72%) | 1.28 (1.23, 1.33) <0.001 |
| PVD, n (%) | 3905 (14.00%) | 1.32 (1.25, 1.40) <0.001 |
| CVD, n (%) | 3905 (14.00%) | 1.18 (1.12, 1.25) <0.001 |
| RD, n (%) | 1135 (4.07%) | 1.03 (0.94, 1.14) 0.492 |
| PUD, n (%) | 1265 (4.54%) | 0.97 (0.89, 1.07) 0.565 |
| Renal Disease, n (%) | 7790 (27.94%) | 1.37 (1.32, 1.43) <0.001 |
| HR (bpm) | 91.61 ± 17.54 | 1.01 (1.00, 1.01) <0.001 |
| SBP (mmHg) | 110.67 ± 13.45 | 0.99 (0.99, 0.99) <0.001 |
| DBP (mmHg) | 60.15 ± 9.64 | 0.98 (0.98, 0.98) <0.001 |
| RR (bpm) | 21.40 ± 4.52 | 1.04 (1.04, 1.05) <0.001 |
| T (℃) | 36.96 ± 0.66 | 0.68 (0.66, 0.70) <0.001 |
| SPO2 (%) | 96.60 ± 2.63 | 0.92 (0.92, 0.93) <0.001 |
| Calcium (mg/dL) | 7.73 ± 0.95 | 1.01 (0.99, 1.03) 0.527 |
| ALB | 2.70 ± 0.88 | 0.96 (0.94, 0.98) <0.001 |
| BUN (mg/dL) | 26.00 (15.00-43.00) | 1.01 (1.01, 1.01) <0.001 |
| Creatinine (mg/dL) | 1.20 (0.80-2.10) | 1.11 (1.10, 1.12) <0.001 |
| GCS score | 9.00 (5.00-14.00) | 0.96 (0.96, 0.97) <0.001 |
| SOFA score | 7.98 ± 4.07 | 1.13 (1.12, 1.13) <0.001 |
| SAPSII score | 46.84 ± 15.86 | 1.03 (1.03, 1.04) <0.001 |
| PTL (109/L) | 212.06 ± 129.44 | 1.00 (1.00, 1.00) <0.001 |
| RBC (1012/L) | 3.54 ± 0.81 | 0.84 (0.82, 0.86) <0.001 |
| WBC (109/L) | 14.53 ± 11.97 | 1.00 (1.00, 1.00) <0.001 |
| Glucose (mg/dL) | 158.00 ± 108.99 | 1.00 (1.00, 1.00) 0.195 |
| Cardiac surgery, n (%) | 6725 (24.12%) | 1.08 (1.04, 1.13) <0.001 |
| Diabetes, n (%) | 10690 (38.34%) | 1.02 (0.98, 1.06) 0.350 |
| Hypertension, n (%) | 15980 (57.31%) | 1.08 (1.04, 1.12) <0.001 |
| MI, n (%) | 5690 (20.41%) | 1.40 (1.34, 1.46) <0.001 |

CI, confidence interval; HR, hazard ratios.

**Table S2 Collinearity screening**

| Variable | Step 1 | Step 2 |
| --- | --- | --- |
| Gender | 1.9 | 1.9 |
| Age (years) | 1.8 | 1.8 |
| Race | 1.1 | 1.1 |
| LOS-ICU (day) | 1.2 | 1.2 |
| Admission BMI (kg/m2) | 4671241.2 | NA |
| Discharge BMI (kg/m2) | 4537589.6 | 57.4 |
| BMI change rate (%) | 11.1 | 11.1 |
| CHF, n (%) | 1.3 | 1.3 |
| PVD, n (%) | 1.1 | 1.1 |
| CVD, n (%) | 1.1 | 1.1 |
| RD, n (%) | 1 | 1 |
| PUD, n (%) | 1 | 1 |
| Renal Disease, n (%) | 1.6 | 1.6 |
| HR (bpm) | 1.4 | 1.4 |
| SBP (mmHg) | 2.7 | 2.7 |
| DBP (mmHg) | 6.6 | 6.6 |
| RR (bpm) | 1.2 | 1.2 |
| T (℃) | 1.2 | 1.2 |
| SPO2 (%) | 1.2 | 1.2 |
| Calcium (mg/dL) | 1.2 | 1.2 |
| ALB | 1.1 | 1.1 |
| BUN (mg/dL) | 1.9 | 1.9 |
| Creatinine (mg/dL) | 2 | 2 |
| GCS score | 1.4 | 1.4 |
| SOFA score | 2.6 | 2.6 |
| SAPSII score | 2.7 | 2.6 |
| PTL (109/L) | 1.2 | 1.2 |
| RBC (1012/L) | 1.2 | 1.2 |
| WBC (109/L) | 1.1 | 1.1 |
| Glucose (mg/dL) | 1.2 | 1.2 |
| Cardiac surgery, n (%) | 1.2 | 1.2 |
| Diabetes, n (%) | 1.3 | 1.3 |
| Hypertension, n (%) | 1.1 | 1.1 |
| MI, n (%) | 1.2 | 1.2 |

**Table S3. Associations between BMI change rate and risk of 30-day mortality based on the raw (non-imputed) dataset.**

| group | Exposure | Number | Event (%) | Model I (HR,95%CI) P | Model II (HR,95%CI) P | Model III (HR,95%CI) P |
| --- | --- | --- | --- | --- | --- | --- |
| All  (N=5577) | BMI change | 5577 | 2068 (37.08) | 1.03 (1.02, 1.03) <0.001 | 1.02 (1.02, 1.03) <0.001 | 1.02 (1.01, 1.02) <0.001 |
|  | Decrease in BMI | 1646 | 461 (28.01) | 1(Ref) | 1(Ref) | 1(Ref) |
|  | Stable BMI | 1883 | 727 (38.61) | 1.63 (1.45, 1.83) <0.001 | 1.59 (1.41, 1.79) <0.0001 | 1.52 (1.38, 1.64) <0.001 |
|  | Increase in BMI | 2048 | 880 (42.97) | 1.74 (1.55, 1.94) <0.001 | 1.65 (1.47, 1.85) <0.001 | 1.56 (1.29, 1.72) <0.001 |

Model I: we did not account for additional variables.

Model II: we adjusted gender, age and race.

Model III: we adjusted gender, age, race, LOS-ICU, CHF, renal disease, PVD; CVD; HR; SBP; RR; T; SPO2; ALB; BUN; Creatinine; SOFA; GCS; SAPSII; PLT; RBC; Cardiac surgery; Hypertension; MI. HR, Hazard Ratios; CI, confidence; Ref, reference.

**Table S4** **Discriminative Ability of Anthropometric Indices for 30-Day Mortality in ICU Sepsis**

| Variable | AUC | (95%CI) |
| --- | --- | --- |
| Admission weight | 53.91% | (53.21% ~ 54.61%) |
| Discharge weight | 51.29% | (50.58% ~ 51.99%) |
| Admission BMI | 53.48% | (52.78% ~ 54.17%) |
| Discharge BMI | 50.32% | (49.61% ~ 51.02%) |
| BMI change | 58.28% | (57.60% ~ 58.95%) |
| BMI change rate | 58.40% | (57.71% ~ 59.08%) |


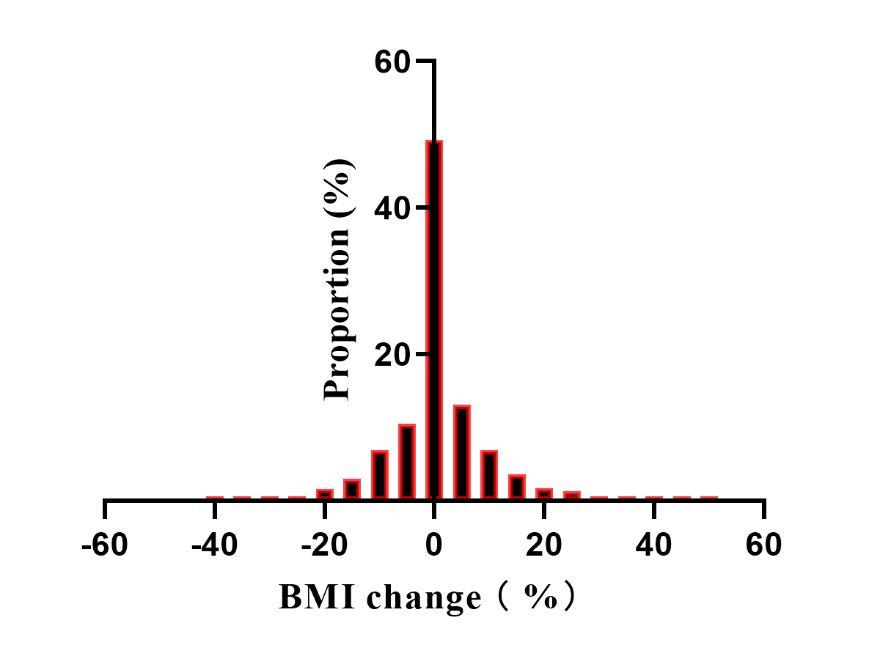


**Figure S1.** Distribution of BMI change (%). It presented a normal distribution, ranging from -39% to 49%, with a mean of 0.85.


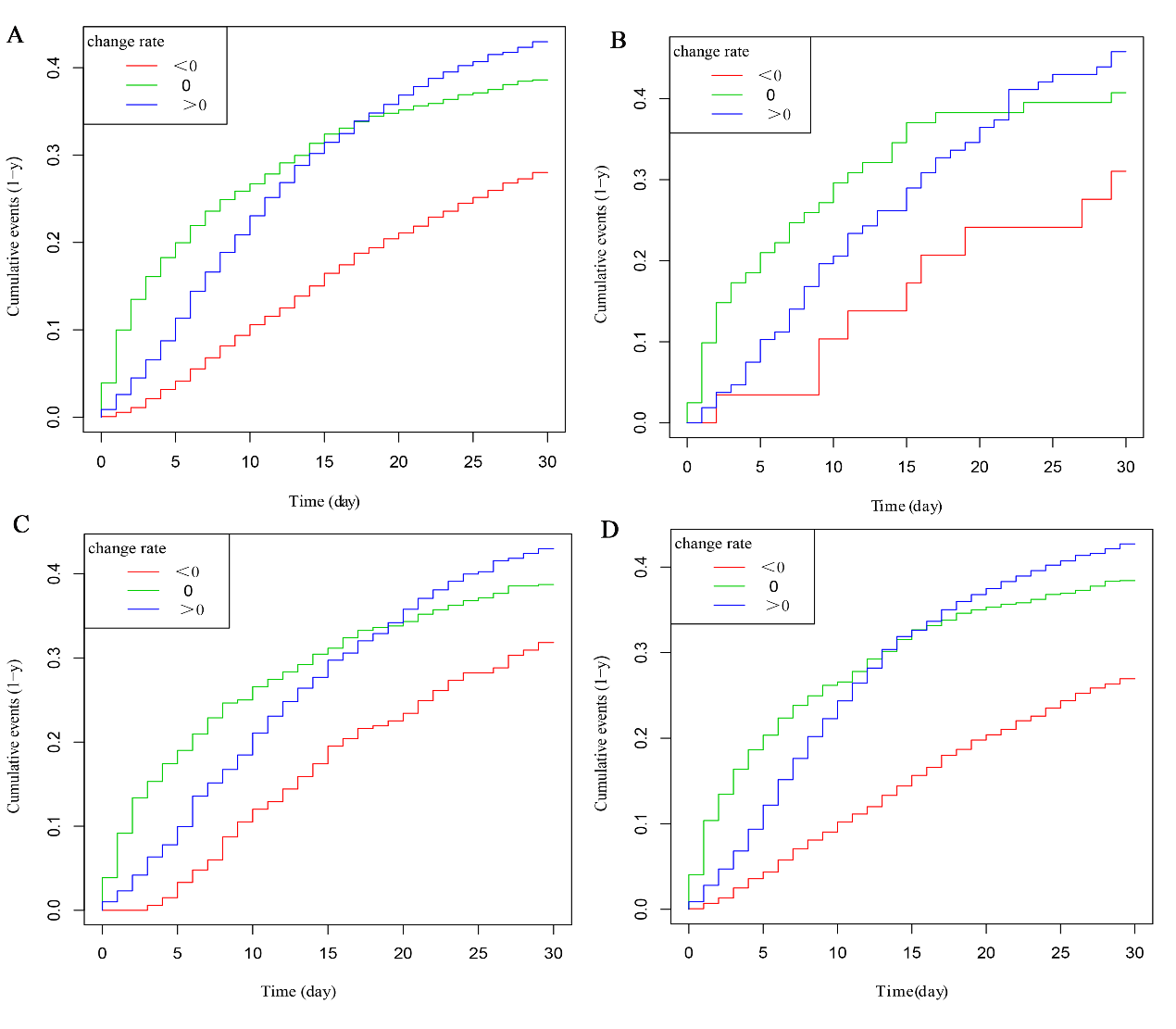


**Figure S2.** Kaplan–Meier estimates of 30-day mortality according to BMI change in ICU sepsis patients.(A) Entire cohort; (B) Baseline BMI <18.5 kg/m² (underweight); (C) Baseline BMI 18.5–24.9 kg/m² (normal weight); (D) Baseline BMI ≥25 kg/m² (overweight/obese).
In each pane l, the red line represents patients with a decrease in BMI during ICU stay; the green line represents patients with stable BMI; the blue line represents patients with increased BMI. The shaded areas indicate 95% confidence bands.


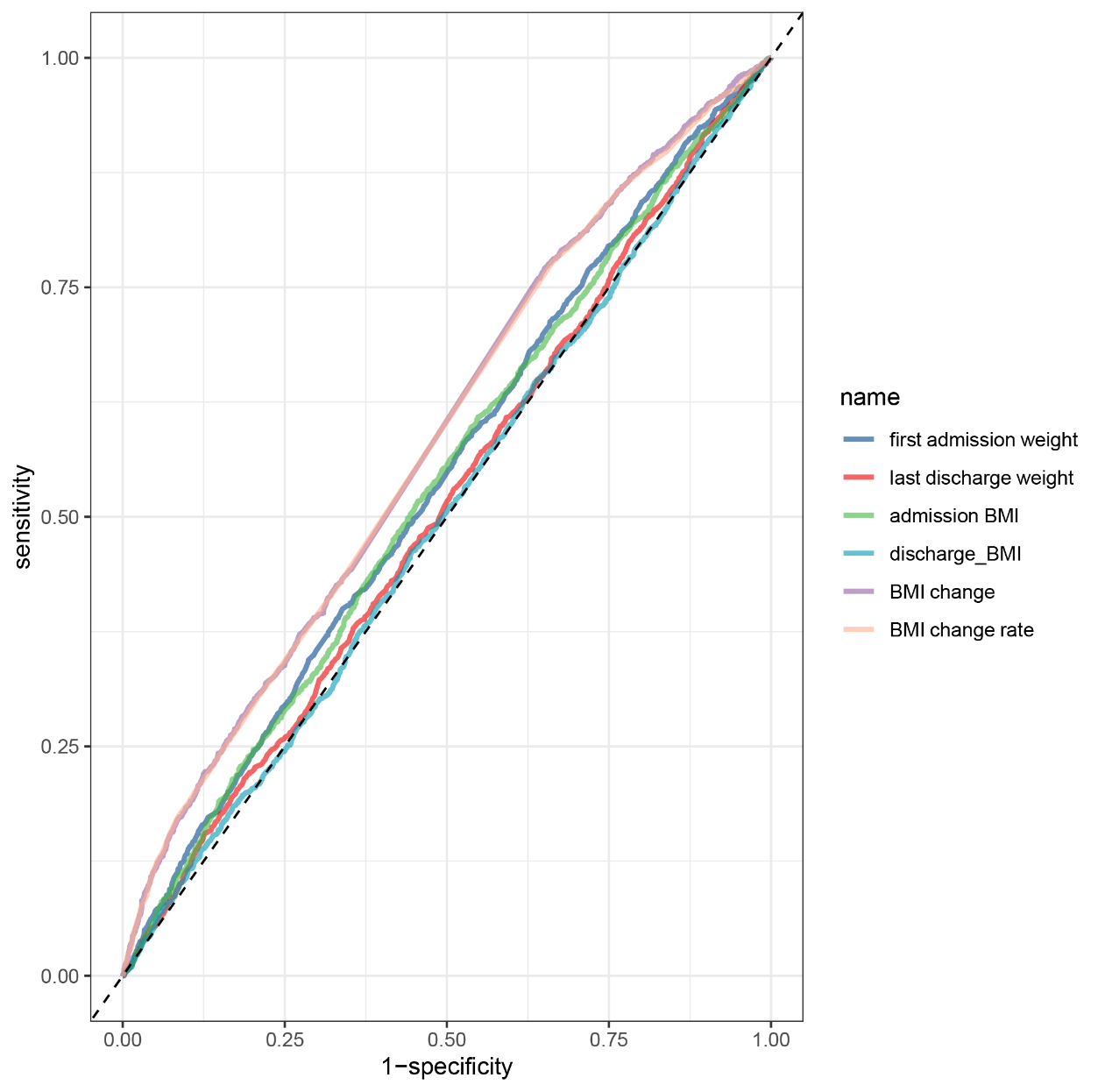


**Figure S3.** Discriminative Performance of Body-Mass Metrics for 30-Day Mortality in ICU Sepsis Patients: Receiver Operating Characteristic Curve Analysis.
